# Pathophysiology of major depressive disorder: mechanisms involved in etiology are not associated with clinical progression

**DOI:** 10.1038/tp.2015.137

**Published:** 2015-09-29

**Authors:** J Verduijn, Y Milaneschi, R A Schoevers, A M van Hemert, A T F Beekman, B W J H Penninx

**Affiliations:** 1 Department of Psychiatry, EMGO Institute for Health and Care Research and Neuroscience Campus Amsterdam, VU University Medical Center, Amsterdam, The Netherlands; 2 Department of Psychiatry, University Medical Center Groningen, University of Groningen, Groningen, The Netherlands; 3 Department of Psychiatry, Leiden University Medical Center, Leiden, The Netherlands

## Abstract

Meta-analyses support the involvement of different pathophysiological mechanisms (inflammation, hypothalamic–pituitary (HPA)-axis, neurotrophic growth and vitamin D) in major depressive disorder (MDD). However, it remains unknown whether dysregulations in these mechanisms are more pronounced when MDD progresses toward multiple episodes and/or chronicity. We hypothesized that four central pathophysiological mechanisms of MDD are not only involved in etiology, but also associated with clinical disease progression. Therefore, we expected to find increasingly more dysregulation across consecutive stages of MDD progression. The sample from the Netherlands Study of Depression and Anxiety (18–65 years) consisted of 230 controls and 2333 participants assigned to a clinical staging model categorizing MDD in eight stages (0, 1A, 1B, 2, 3A, 3B, 3C and 4), from familial risk at MDD (stage 0) to chronic MDD (stage 4). Analyses of covariance examined whether pathophysiological mechanism markers (interleukin (IL)-6, C-reactive protein (CRP), cortisol, brain-derived neurotrophic factor and vitamin D) showed a linear trend across controls, those at risk for MDD (stages 0, 1A and 1B), and those with full-threshold MDD (stages 2, 3A, 3B, 3C and 4). Subsequently, pathophysiological differences across separate stages within those at risk and with full-threshold MDD were examined. A linear increase of inflammatory markers (CRP *P*=0.026; IL-6 *P*=0.090), cortisol (*P*=0.025) and decrease of vitamin D (*P*<0.001) was found across the entire sample (for example, from controls to those at risk and those with full-threshold MDD). Significant trends of dysregulations across stages were present in analyses focusing on at-risk individuals (IL-6 *P*=0.050; cortisol *P*=0.008; vitamin D *P*<0.001); however, no linear trends were found in dysregulations for any of the mechanisms across more progressive stages of full-threshold MDD. Our results support that the examined pathophysiological mechanisms are involved in MDD’s etiology. These same mechanisms, however, are less important in clinical progression from first to later MDD episodes and toward chronicity.

## Introduction

Several biological mechanisms with a possible role in major depressive disorder (MDD)’s pathophysiology have been identified, and it has been hypothesized that these mechanisms may have a role both in the etiology and progression of the disorder. Here, we consider four central mechanisms that have substantial support in literature for their importance in depression etiology and that have previously been shown to be significantly different in MDD cases and controls in our own sample: inflammation,^1^ hypothalamic–pituitary (HPA) axis,^2^ neutrophic growth^3^ and vitamin D.^4^ We examined whether progression of MDD at a clinical level (that is, that what patient/clinicians experience as more advanced disease stage, which is multiple episodes and/or chronicity) is paralleled by more pronounced dysregulation in pathophysiological mechanisms, as evidence for that is scarce.

An upregulation of inflammation might be involved in the development of depression by decreasing the production of monoamines (for example, serotonin), and increasing the production of tryptophan catabolites that are toxic for the brain.^5^ Meta-analyses showed that depressed subjects in comparison with controls had significantly increased levels of the pro-inflammatory cytokine interleukin (IL)-6,^6, 7, 8^ and acute phase C-reactive protein (CRP).^6^ An example of inflammation and MDD progression is the finding that tumor necrosis factor alpha was significantly higher in those who experienced >3 episodes.^5^

Hyperactivity of the HPA-axis as a causal factor in MDD has been studied extensively.^9^ This hyperactivity is presumably caused by malfunctioning of glucocorticoid receptors impairing the negative feedback circuit of the HPA-axis. Glucocorticoid receptor malfunction might cause depression via impaired neurogenesis and reduced hippocampus volumes.^10, 11^ In depressed persons, cortisol levels might determine risk of^12^ and time to recurrence^13^ of an MDD episode. This suggest that HPA-axis dysfunctioning is associated with MDD progression.

Low levels of brain-derived neurotrophic factor (BDNF) are seen as an indicator of reduced neurotropic growth, a third important possible pathophysiological mechanism of MDD.^14^ A recent meta-analysis supports the idea that depressed patients have lower BDNF levels than controls.^15^ One study found that drug-free patients with a long index episode had significantly lower BDNF levels compared with patients with a shorter index episode.^16^

Recently low levels of vitamin D have been associated with depression in a meta-analysis.^17^ Different pathophysiological mechanisms have been suggested via which vitamin D might be involved with the etiology or progression of depression.^18, 19^ For instance, vitamin D might be neuroprotective^20^ by reducing neurotoxic calcium levels in the brain.^21^ To our knowledge, no studies exist that examined whether vitamin D is associated with the progression of depression.

The majority of previous studies were based on case–control comparisons, generally with highly heterogeneous groups with a different developmental history of depression in terms of severity, duration or number of experienced disease episodes. Examining differences in the pathophysiological mechanisms across groups of patients subdivided according to their developmental depression trajectory, for instance done by clinical staging, provides the opportunity to test whether mechanisms that are important for the etiology of depression are also important in progression of MDD at a clinical level toward multiple episodes and/or chronicity. Clinical staging is a tool to describe progression at a clinical level; it aims to divide the natural course of the disorder in clinically detectable phases that reflect disease progression and that possess clinical significance for prognosis and choice of treatment.^22^ Ultimately staging may contribute to early recognition of MDD development and lead to early intervention that prevents progression to later stages.^23^ Staging has proven useful in the fields of schizophrenia^24, 25^ and bipolar disorder.^26^ Currently, the most used staging model for MDD^27^ consists of eight stages. Three at-risk for MDD stages that are concerned with the initial phases of MDD development and five full-threshold MDD stages that reflect progression of MDD (Supplement Table 1). The staging model serves two purposes: (i) it puts the focus on early recognition of healthy people at risk for depression; (ii) people with a full-threshold disorder are assigned to consecutive stages of disease progression based on number and duration of the current episode, reflecting treatment necessity and prognosis.

**Figure 1:**
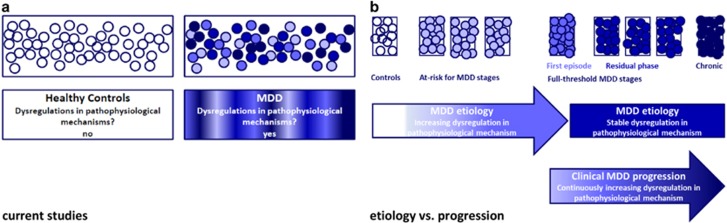
Assumptions of this study. Current studies examined the association between pathophysiological mechanism in a heterogeneous group of MDD patients (**a**). We studied the association between pathophysiological mechanisms and groups of MDD patients divided according to the developmental course of the disorder using staging. We hypothesized that the pathophysiological mechanisms would be associated with both etiology and clinical progression of MDD. Therefore, we expected increasing dysregulations of the pathophysiological mechanisms across all the consecutive stages (progression, **b**). MDD, major depressive disorder.

We examined whether progression of MDD at a clinical level was paralleled by more pronounced dysregulations in four central pathophysiological mechanisms. We used staging to define clinical progression of MDD. The pathophysiological mechanisms were studied via markers that reflect those mechanisms. In a large cohort, well characterized in terms of psychiatric diagnoses and clinical characteristics of MDD, we compared levels of inflammatory markers, HPA-axis hormones, BDNF and vitamin D across healthy controls (HCs), subjects in at-risk for MDD stages and full-threshold MDD stages. Using this approach, we could test two assumptions for our study (see Figure 1). First, pathophysiologic mechanisms are involved in the etiology but are not associated with clinical progression of MDD. In this case, we expect the markers to show an increasing gradient of dysregulation across ascending at-risk stages, which stabilizes across full-threshold stages. Second, the mechanisms are associated with clinical progression of MDD as well. If so, we expect the markers to show continuously increasing dysregulations across consecutive full-threshold stages. We hypothesized to find evidence for the second assumption, as a few previous studies^5^ suggest a role of these mechanisms beyond etiology.

## Materials and methods

### Sample

Participants were selected from the Netherlands Study of Depression and Anxiety (NESDA), an on-going cohort study into the etiology and prognosis of depressive and anxiety disorders. At baseline, 2981 adults (18–65 years) were selected from community (19%), general practice (54%) and specialized mental health care (27%) to represent the entire developmental spectrum of both disorders, including HCs. Uniform inclusion and exclusion criteria were used across recruitment settings and exactly similar measurement procedures, conducted by the same research staff, were followed in order to achieve uniform assessments. The methodology of NESDA has been extensively described elsewhere.^28^ The ethical review boards of contributing universities approved the study, and all participants signed informed consent. At baseline, participants gave blood samples and underwent a physical examination and a psychiatric interview. The presence of a depressive (MDD and/or dysthymia) and/or anxiety disorder (social phobia, agoraphobia, panic- and/or generalized anxiety disorder) was assessed with the DSM-IV Composite International Diagnostic Interview (CIDI) version 2.1.^29^ The number of MDD episodes experienced was extracted from the CIDI. The severity of depression was measured with the Inventory of Depressive Symptoms (IDS).^30^ The duration of depressive symptoms was examined with the Life Chart,^31^ calculated as the number of months with depressive symptoms in the 3 years prior to the baseline interview.

For the current study, we selected 2563 subjects of the baseline sample who were either HCs or assigned to one of the eight stages of MDD. HCs (*n*=230) were without a lifetime diagnosis of MDD or anxiety disorder, without depressive symptoms (IDS≤13) and without a first-degree family member with depression. The assignment of subjects to MDD stages is described in our previous paper^32^ and can be found in Supplement Table 1. In short, the assignment to stages is based on the lifetime presence of an MDD and/or anxiety disorder, the recency of an MDD episode (current if present in the 6 months prior to baseline, otherwise remitted), severity and duration of depressive symptoms and the number of episodes. The at-risk stages consisted of subjects without lifetime MDD or anxiety diagnosis, but with: stage 0 (*n*=287) a first-degree family member with depression; stage 1A (*n*=116) mild depressive symptoms; stage 1B (*n*=834) sub-threshold depressive symptoms and subjects with remitted MDD. Full-threshold stages included patients with a current MDD episode: stage 2 (*n*=230) first MDD episode; stage 3A (*n*=129) incomplete remission of first episode; stage 3B (*n*=127) recurrence or relapse of MDD; stage 3C (*n*=394) multiple relapses; stage 4 (*n*=216) chronic MDD.

### Pathophysiological mechanism markers

#### Inflammatory markers

As described in more detail elsewhere,^1^ CRP and IL-6 were assayed at the Clinical Chemistry Department of the VU University Medical Centre from fasting blood samples obtained in the morning and kept frozen at −80 °C. High-sensitivity plasma levels of CRP were measured in duplicate by an in-house enzyme-linked immunosorbent assay based on purified protein and polyclonal anti-CRP antibodies (Dako, Glostrup, Denmark). Plasma IL-6 levels were measured in duplicate by a high-sensitivity enzyme-linked immunosorbent assay (PeliKine Compact ELISA, Sanquin, Amsterdam, The Netherlands). Intra- and inter-assay coefficients of variation were 5 and 10% for CRP, and 8% and 12% for IL-6, respectively.

A previous NESDA study^1^ found that currently depressed men, but not women, had higher levels of CRP (*d*=0.21) and IL-6 (*d*=0.10) than nondepressed peers. We selected 2526 (98.6%) participants with at least one available inflammatory marker. As the previous NESDA study found gender-specific associations between depression and inflammatory markers, we stratified all inflammation analyses by gender.

#### HPA-axis markers

As described in more detail elsewhere,^2^ participants collected saliva samples at home shortly after the baseline interview. Saliva samples were obtained using Salivettes (Sarstedt, Nümbrecht, Germany) at seven time points. We used four composite HPA-axis markers (i) the area under the curve with respect to the ground (AUCg, measure of the total morning cortisol secretion); and (ii) the area under the curve with respect to the increase (AUCi, measure of cortisol dynamic) were calculated if all four morning samples (T1=awakening time, T2=30, T3=45, T4=60 min later) were available by use of trapezoid formulas;^33^ (iii) evening cortisol (the mean of evening levels T5 at 2200 hours and T6 at 2300 hours); and (iv) the cortisol suppression ratio (a ratio obtained by dividing the cortisol value at awakening on the first day (T1) by the cortisol value at awakening on the next day (T7) after ingestion of 0.5 mg dexamethasone. This dexamethasone suppression test examines the functioning of the negative HPA-axis feedback mechanism. Our analyses included only those participants (*n*=1723, 67.2%) who had information on at least one of the four HPA-axis markers. Previous NESDA results^2^ showed that when compared with HCs, both current and remitted depressed participants had a (significantly) higher AUCg and AUCi, higher evening cortisol levels at 2200 hours, but not a different cortisol suppression ratio.

#### BDNF

As described in more detail elsewhere,^34^ serum was separated immediately after blood draw and stored (at −85 °C) until assay. Serum BDNF concentrations were determined using the Emax Immuno Assay system from Promega according to the manufacturer’s protocol (Madison, WI, USA). Absorbency was read in duplicate using a Bio-Rad Benchmark microplate reader (Hercules, CA, USA) at 450 nm. The coefficients of variance ranged between 2.9 and 8.1%. In total, 2498 (97.5%) of the subjects included in our sample had a BDNF value available. A previous NESDA paper^3^ showed that anti-depressant-free currently depressed participants had lower BDNF levels than both HCs and their medicated currently depressed peers.

#### Vitamin D

As described in more detail elsewhere,^4^ vitamin D was measured by assessing the blood’s circulating levels of 25(OH)D, which is the combined product of cutaneous synthesis from solar exposure and dietary sources. Serum 25(OH)D was measured using isotope dilution–online solid-phase extraction liquid chromatography–tandem mass spectrometry (ID-XLC-MS/MS).^35^ Intra- and inter-assay coefficients of variation were <6% and <8%, respectively, for concentrations between 25 and 180 nmol l^−1^. In our sample, 2514 (98.1%) subjects had a vitamin D value. A previous NESDA study^4^ showed that compared with controls, both currently depressed and remitted depressed had lower vitamin D levels.

### Covariates

Putative covariates were selected *a priori* based on previous analyses and divided into general covariates and covariates specific per marker. General covariates included socio-demographic variables age (years), gender (male/female) and education (years), and health indicators smoking status (never, former and current), alcohol use (non-drinker <1 units per week; mild/moderate drinker female 1–14, male 1–21 units per week; heavy drinker female >14 and male >21 units per week),^36^ number of self-reported chronic diseases for which the subject received treatment (including cardiovascular diseases, diabetes, lung disease, arthritis, cancer, ulcer, intestinal problem, liver disease, epilepsy and thyroid gland disease), and body mass index expressed in kg/m^2^. Participants were asked to bring their medication packages to the interview after which medications were registered according to World Health Organization Anatomical Therapeutic Chemical classification.^37^ Covariates specific per marker included (1) for inflammation, the use of systemic anti-inflammatory drugs (M01A, M01B, A07EB and A07EC); (2) for the HPA-axis, as reported on the cortisol sampling form the factors awakening time (hh:mm), work status (yes/no) and season of saliva collection (light/dark); (3) for BDNF, the use of systemic anti-inflammatory drugs, non-opioid analgesic-antipyretics (N02BA and N02BE) and selective serotonin reuptake inhibitors (SSRI and N06AB); (4) for vitamin D, the sampling factor season of blood collection (light/dark). Season during saliva and blood collection were categorized in light months (March–September) and dark months (October–February). Inflammation, HPA-axis and vitamin D were not adjusted for antidepressant use, as previous NESDA studies^1, 2, 4^ showed that antidepressant use was not strongly associated with these pathophysiological markers, nor did they change the association found between depression and markers. In depressed persons, SSRI users had higher BDNF levels than non-SSRI users,^3^ therefore we adjusted our BDNF analyses for SSRI use.

### Statistical analyses

Differences in demographic variables and covariates between HCs, the at-risk for MDD group (stages 0 through 1B) and the full-threshold MDD group (stages 2 through 4) were examined using analyses of variance and the *χ*^2^-test, followed by Games-Howell *post hoc* tests.

We compared levels of pathophysiological markers across subjects assembled using two different strategies (A and B). In strategy A, we contrasted the three main groups, namely, HCs, at-risk group (stages 0, 1A, and 1B combined) and full-threshold group (stages 2, 3A, 3B, 3C and 4 combined). In strategy B, we contrasted the separate stages (HCs, 0, 1A, 1B, 2, 3A, 3B, 3C and 4). For both strategy A and B, we conducted two analyses. First differences in adjusted means of the pathophysiological markers across the groups/stages were tested with analyses of covariance, followed by Tukey's Least Significant Difference (Tukey's LSD) *post hoc* pairwise comparisons. Second, we tested whether the biological measure showed a linear trend across the groups/stages. For strategy B (separate stages), we performed a third analysis. To examine whether a trend across all separate stages was caused by differences within at-risk stages and/or within full-threshold stages, trend analyses were repeated focusing on either the controls-at-risk stages (HCs, 0, 1A and 1B) or the full-threshold stages (2, 3A, 3B, 3C and 4).

Finally, we examined whether possible differences in pathophysiological markers across full-threshold stages could be explained by any of the more specific clinical characteristics that were used to create the MDD staging model: severity (continuous IDS score), duration (% of time with depressive symptoms in 3 years before baseline) and number of episodes. Analyses were additionally adjusted for socio-demographic factors (model 1), health indicators (model 2) and covariates specific per biological measure (model 3). Pathophysiological markers that were non-normally distributed (inflammatory markers, mean evening cortisol and cortisol suppression ratio) were log-transformed before analysis and presented back transformed. All analyses were performed with SPSS version 20.0. (SPSS, Chicago, IL, USA) Significance was set a *P*<0.05, using two-tailed tests.

## Results

Table 1 shows the socio-demographic and health-related sample characteristics, and pathophysiological markers across HCs, the at-risk group and the full-threshold MDD group. As compared with controls, the at-risk and the full-threshold group were more often women and current smokers, had lower education, more chronic diseases and were more likely to use SSRI antidepressants or anti-inflammatory medication. Moreover, the full-threshold and at-risk groups as compared with HCs had higher CRP and cortisol levels and lower vitamin D.

### Comparing pathophysiological mechanism markers

#### Between HCs, the at-risk for MDD group and full-threshold MDD group

Inflammatory markers: In men, we found a significant linear trend across the three main groups for both CRP and IL-6 (Table 2), even after full adjustment. The full-threshold MDD group had significantly higher CRP levels than both HCs and the at-risk group, and had significantly higher IL-6 levels than HCs. In women, no such associations were found.

HPA-axis: Both measures of the cortisol awakening response (AUCg and AUCi) were higher in the at-risk and full-threshold MDD groups when compared with controls. After full adjustment, AUCg showed an upward linear trend, but AUCi did not. Mean evening cortisol and the cortisol suppression ratio did not show a linear trend across groups after full adjustment.

BDNF: Unadjusted and fully adjusted analyses did not show a trend of BDNF levels across the three main groups.

Vitamin D: Both unadjusted and adjusted vitamin D levels showed a highly significant linear trend across the three main groups, with the at-risk group having significant lower vitamin D levels than the HCs and the full-threshold MDD group having significant lower vitamin D levels than the at-risk group.

#### Between separate stages

Inflammatory markers: In both men and women, CRP levels showed a significant linear trend across the separate stages from controls through stage 4, but no trend existed across the controls/at-risk stages from controls through stage 1B, or across full-threshold stages 2 through 4 (Table 3). Furthermore, in both men and women IL-6 levels showed no significant trend across all stages. However, in women across controls/at-risk stages a significant downward trend was found.

HPA-axis: AUCg showed a significant linear trend across the separate stages, a highly significant trend across HCs/at-risk stages, but no trend across full-threshold stages. AUCi only showed a linear trend across the HCs/at-risk stages. The lowest levels of AUCg and AUCi were for stage 1A, those without a history of MDD but mild symptoms. The highest levels were in stage 3A, those with an incomplete remission from a first episode. After full adjustment, no linear trend across the stages was found for the mean evening cortisol or for the cortisol suppression ratio.

BDNF: Unadjusted and fully adjusted analyses did not show any linear trend for BDNF across stages.

Vitamin D: After full adjustment, vitamin D showed a significant downward linear trend across the separate stages, meaning that later stages had lower vitamin D levels. However, follow-up trend analyses showed a significant downward linear trend across HCs/at-risk stages, but not across the full-threshold stages.

#### Full-threshold MDD stages explained by separate clinical characteristics

As we hardly found any indication that pathophysiological dysregulation was larger across more progressive MDD stages, we checked whether indeed associations were also absent for specific MDD clinical characteristics. Within full-threshold MDD patients, only two of the 30 tested associations between characteristics (severity-, duration- and number of episodes) and the biological markers were (borderline) significant (Table 4). Severity of depression showed a significant negative association with the AUCg level (*β*=−0.086, s.e.=0.040, *P*=0.034), and the number of episodes showed a borderline significant association with CRP in women (*β*=−0.067, s.e.=0.034, *P*=0.050).

## Discussion

The present study examined whether progression of MDD at a clinical level (defined by clinical staging) was paralleled by more advanced pathophysiological dysregulations. We examined four central pathophysiological mechanisms (inflammation, HPA-axis, neurotrophic growth and vitamin D). We hypothesized that besides involvement in the etiology of MDD, these mechanisms are also associated with clinical progression of MDD, as reflected by continuously increasing dysregulation across consecutive full-threshold stages. That hypothesis was not upheld. Three (inflammation, HPA-axis and vitamin D) of the four examined pathophysiological mechanisms showed increasing trends of dysregulation across HCs and the at-risk stages (0, 1A and 1B) of depression, but not across the full-threshold stages (2, 3A, 3B, 3C and 4). These results suggest that mechanisms involved in the etiology of depression are not associated with clinical progression of depression.

Our finding that the pathophysiological mechanisms are involved in the etiology of depression confirms previous meta-analysis^6, 7, 8, 9, 15, 17^ and longitudinal studies.^12, 38, 39, 40, 41^ Interestingly, we did not find any association between BDNF and MDD groups/stages, which is in contrast with previous studies that found lower BDNF levels in untreated currently depressed patients that normalize in treated patients.^15^ However, our results are in line with more recent studies that showed that the association between BDNF and MDD etiology is still unclear and modest at the best; moreover, BDNF seems rather to modulate the treatment efficacy.^42^

We have tested the hypothesis that the examined mechanisms could also be associated with clinical progression of MDD. Although this intriguing research hypothesis generates mainly from data on animal studies,^5^ the few observational studies on humans supporting it included small sample sizes,^43^ consisted of very selective samples^12, 44, 45^ or only examined one aspect of depression progression, for example, recurrence.^13^ To our knowledge, this is the first study that, within a large sample, tested whether pathophysiological mechanisms show more dysregulation in clinically more progressed depression, as defined by the tool clinical staging. Using this approach, we found that the examined mechanisms are not more dysregulated in clinically progressed MDD. Moreover, when we examined whether the separate clinical measures (severity, duration and number of episodes) that build up the staging model were associated with more dysregulation in the pathophysiological mechanism, we did not find an association either. Together these results strongly suggest that the examined mechanisms are not more dysregulated in clinically progressed MDD. When the examined mechanisms would have shown more dysregulation in clinically progressed MDD, the mechanisms could have contributed to an index to separate different stages of MDD progression (=biomarker of stage^46^). The clinical utility of using pathophysiological markers as an objective measure to separate stages of disease progression is evident from somatic disorders. For instance, kidney failure knows five stadia based on the estimated glomerular filtration rate, inter alia based on serum creatinine level,^47^ a relatively easy and very objective measure. Stages of disease progression have clinical significance for prognosis and choice of therapeutic modality.^22^ For example, it has helped to make the case for early detection and to devise specific treatments for specific stages of kidney failure.^47^ It has been hypothesized that application of staging to MDD may have similar benefits.^48, 49^ However, the here examined mechanisms cannot be used as an index to separate different stages of MDD progression. It could be, however, that other pathophysiological mechanisms are more dysregulated in clinically progressed MDD. For instance, besides the mechanisms we studied for progression, Moylan *et al.*,^5^ suggest inter alia neurotransmitter systems, oxidative and nitrosative stress, mitochondrial dysfunction and epigenetic influences. Furthermore, it could be that the examined mechanisms are involved in disease progression only in certain subtypes of depression, or only in patients with certain clinical characteristics. For example, previous research has shown that upregulation of inflammation is mainly present in those with atypical depression, and hypercortisolemia in those with melancholic depression.^50^ Those that experienced a childhood trauma had instead lower cortisol levels.^13, 51, 52^ Moreover, as recently well described by Davis *et al.*^46^ it might be that the examined mechanism markers are not biomarkers of stage, but are biomarkers of diagnosis/trait (inflammation, cortisol and vitamin D) indicating whether in a person a depression is present or not, or biomarkers of treatment response (BDNF) indicating whether an individual is likely to respond to a certain treatment or not, ideally giving the clinician the opportunity to personalize treatments to individual needs. Finally, it is very well possible that clinical progression of MDD is not driven by increased dysregulation of the studied mechanisms, but rather by prolonged exposure to a chronic level of dysregulation that then leads to damage/alterations of cellular components, which subsequently could cause a person to be at further risk of recurrence or chronicity of MDD.

The main strengths of our study are the large number of well-diagnosed patients in the whole adult age range representing different developmental stages of MDD, and the availability of a wide range of important confounders regarding the examined pathophysiological mechanisms. However, our study also has some limitations. First, biological measurements were obtained via a blood draw or saliva collection, which are peripheral measurements that may not necessarily represent the ‘central/brain’ mechanism relevant for depression. However, they have been consistently associated with MDD status in previous studies.^6, 7, 8, 9, 15, 17^ Furthermore, whether the measured markers are the best indexes for the candidate mechanisms of depression pathophysiology has not been definitely established. Second, we used a staging model to define clinical progression of MDD in a cross-sectional sample. To make a definite statement about involvement of the mechanisms in clinical progression of MDD, a longitudinal study would be necessary in which markers are preferably measured before onset of depression, during and after the first and follow-up episodes. Third, we used the most applied staging model for MDD that stages patients mainly according to disease episode number and duration. When we separately tested the clinical characteristics of severity, duration and number of episodes, none of them were consistently associated with the measured mechanism markers. It might be that aspects of disease that were omitted in the staging model, such as complications, functional outcome and comorbidity, are of more importance for clinical MDD progression. Indeed some studies showed an association between the pathophysiological dysregulations and suicidal ideation (inflammation^53^ and HPA^54^) and comorbidity (inflammation,^55^ HPA^56^ and vitamin D^57^). Finally, we like to acknowledge that different staging models for MDD exist,^58^ besides the one we have chosen to use.^27^ The main difference is that those models collapse stage 3A—incomplete remission of first episode, stage 3B—first relapse and stage 3C—multiple recurrent episodes into one stage three-relapsing/reoccurring MDD. The decision to use the extensive staging model was based on our previous finding that stage 3A in general had worse characteristics than the sublevel 3B and 3C, and scores more similar as stage 4 (chronic MDD).^32^

In conclusion, three candidate pathophysiologic mechanisms (inflammation, HPA-axis and vitamin D) show increased dysregulation across controls and at-risk stages of development of MDD, suggesting involvement in the etiology of MDD. Nevertheless, the same mechanisms did not show more dysregulation in clinical progression of MDD toward multiple episodes and/or chronicity. This suggests that pathophysiological mechanisms for etiology and clinical disease progression are not necessarily overlapping. More (longitudinal) research on pathophysiological mechanisms that drive clinical disease progression is needed in order to be able to use dysregulations in those mechanisms as markers of clinical progression of depression and in such for staging.

## Figures and Tables

**Table 1:** Sample characteristics: healthy controls, at-risk for MDD group and full-threshold MDD group

	*Total sample* n=*2563*	*Healthy controls* n=*230*	*At-risk for MDD group (stages 0, 1A and 1B)* n=*1237*	*Full-threshold MDD group (stages 2, 3A, 3B, 3C and 4)* n=*1096*	P*-value*
*Demographics*					
Age (years), M (s.d.)		43.5 (13.8)	42.4 (13.5)	40.8 (12.1)^hc,ar^	0.002
Gender (female), *n* (%)		132 (57.4)	843 (68.1)	740 (67.5)	0.005
Years of education, M (s.d.)		13.2 (3.1)	12.4 (3.2)^hc^	11.6 (3.2)^hc,ar^	<0.001

*Lifestyle and health factors*					
BMI (kg/m^2^), M (s.d.)		25.7 (4.6)	25.4 (4.7)	25.9 (5.4)^ar^	0.040
Smoking status, *n* (%)					<0.001
Never		92 (40.0)	341 (27.6)	290 (26.5)	
Former		88 (38.3)	449 (36.3)	312 (28.5)	
Current		50 (21.7)	447 (36.1)	494 (45.1)	
Drinking behavior, *n* (%)					<0.001
Non-drinker		54 (23.5)	336 (27.2)	438 (40.0)	
Mild-moderate drinker		147 (63.9)	763 (61.7)	531 (48.4)	
Heavy drinker		29 (12.6)	138 (11.2)	127 (11.6)	
Number of chronic diseases, M (s.d.)		0.45 (0.71)	0.58 (0.85)^hc^	0.69 (0.95)^hc,ar^	<0.001
Pathophysiological mechanism markers and their specific covariates					P*-value*
*Inflammation*	*n*=2526	*n*=228	*n*=1215	*n*=1083	
C-reactive protein (mg l^−1^)^a^, M (s.d.)		1.14 (3.09)	1.21 (3.40)	1.39 (3.59)^hc,ar^	0.010
Interleukin-6 (pg ml^−1^)^a^, M (s.d.)		0.71 (2.47)	0.74 (2.57)	0.80 (2.63)	0.100
Systemic anti-inflammatory med., *n* (%)		2 (0.9)	53 (4.4)	50 (4.6)	0.032

*HPA-axis function*	*n*=1723	*n*=176	*n*=871	*n*=676	
AUCg (nmol l^−1 ^h^−1^), M (s.d.)		18.2 (7.0)	18.9 (6.6)	19.4 (7.4)	0.138
AUCi (nmol l^−1^ h^−1^), M (s.d.)		0.97 (6.47)	2.39 (6.24)^hc^	2.41 (6.26)^hc^	0.022
Mean evening cortisol (nmol l^−1^)^a^, M (s.d.)		4.33 (1.74)	4.65 (1.70)	4.89 (1.74)^hc^	0.019
Cortisol suppression ratio^a^, M (s.d.)		2.45 (1.67)	2.38 (1.63)	2.40 (1.68)	0.764
Mean awakening time (h:min), M (s.d.)		7:17 (1:08)	7:27 (1:02)	7:31 (1:10)^hc^	0.038
Working on day saliva collection, *n* (%)		122 (69.3)	566 (65.0)	384 (56.8)	0.001
Season saliva collection (light), *n* (%)		106 (60.2)	547 (62.8)	404 (59.8)	0.453

*Neurotrophic growth*	*n*=2498	*n*=226	*n*=1204	*n*=1068	
BDNF (ng ml^−1^), M (s.d.)		9.22 (3.01)	9.01 (3.21)	9.00 (3.41)	0.636
Systemic anti-inflammatory med., *n* (%)		2 (0.9)	53 (4.4)	49 (4.6)	0.034
Non-opioid analgesic-antipyretic med., *n* (%)		16 (7.1)	112 (9.3)	107 (10.0)	0.383
Anti-depressant: SSRI, *n* (%)		0 (0.0)	131 (10.9)	319 (29.9)	<0.001

*Vitamin D*	*n*=2514	*n*=228	*n*=1212	*n*=1074	
25(OH)D (nmol l^−1^), M (s.d.)		70.7 (27.4)	64.5 (27.7)^hc^	59.9 (28.5)^hc,ar^	<0.001
Season blood collection (light), *n* (%)		123 (53.9)	717 (59.2)	593 (55.2)	0.102

Abbreviations: ar, at-risk for MDD group; AUCg/i, area under the curve with respect to the ground/increase; BDNF, brain-derived neurotrophic factor; BMI, body mass index; hc, healthy controls; HPA, hypothalamic–pituitary axis; M=mean; MDD, major depressive disorder; med., medication; SSRI, selective serotonin reuptake inhibitors.

a Log-transformed factors presented back-transformed. Superscripts (^hc,ar^) refer to which group this group its outcome differs signficantly (*P*<0.05) from; for example, the full-threshold MDD group is significantly younger compared with both healthy controls and the at-risk for MDD group. Differences between groups were examined using Games-Howell *post hoc* tests.

**Table 2:** Adjusted levels of pathophysiological markers across healthy controls, at-risk for MDD group and full-threshold MDD group

n=*2563*	*Healthy controls* n=*230*	*At-risk for MDD group (stages 0, 1 A and 1B)* n=*1237*	*Full-threshold MDD group (stages 2, 3 A, 3B, 3C and 4)* n=*1096*	P *for trend*
	*Mean (CI)*	*Mean (CI)*	*Mean (CI)*	
*Inflammation*	*n*=228	*n*=1215	*n*=1083	
*Male*	*n*=97	*n*=389	*n*=350	
C-reactive protein (mg l^−1^)^a^				
Model 1	0.90 (0.71–1.12)	1.04 (0.93–1.17)	1.32 (1.17–1.49)^hc,ar^	<0.001
Model 2	1.01 (0.82–1.24)	1.03 (0.93–1.14)	1.30 (1.16–1.45)^hc,ar^	0.004
Model 3	1.01 (0.82–1.25)	1.03 (0.93–1.14)	1.29 (1.16–1.44)^hc,ar^	0.005
Interleukin-6 (pg ml^−1^)^a^				
Model 1	0.63 (0.53–0.76)	0.81 (0.75–0.89)^hc^	0.89 (0.81–0.98)^hc^	0.002
Model 2	0.67 (0.56–0.80)	0.81 (0.74–0.88)	0.88 (0.80–0.97)^hc^	0.012
Model 3	0.67 (0.56–0.81)	0.81 (0.74–0.88)	0.88 (0.80–0.96)^hc^	0.013
*Female*	*n*=131	*n*=826	*n*=733	
C-reactive protein (mg l^−1^)^a^				
Model 1	1.45 (1.17–1.79)	1.35 (1.24–1.47)	1.35 (1.23–1.48)	0.656
Model 2	1.40 (1.15–1.69)	1.42 (1.31–1.53)	1.29 (1.19–1.40)	0.153
Model 3	1.40 (1.15–1.69)	1.42 (1.31–1.53)	1.29 (1.19–1.40)	0.154
Interleukin-6 (pg ml^−1^)^a^				
Model 1	0.79 (0.67–0.92)	0.72 (0.67–0.76)	0.75 (0.69–0.80)	0.939
Model 2	0.79 (0.67–0.92)	0.74 (0.69–0.78)	0.72 (0.68–0.77)	0.403
Model 3	0.78 (0.67–0.92)	0.74 (0.69–0.79)	0.72 (0.67–0.77)	0.405
*HPA-axis*	*n*=176	*n*=871	*n*=676	
AUCg (nmol l^−1^ h^−1^)				
Model 1	18.1 (17.0–19.1)	18.9 (18.4–19.4)	19.4 (18.9–20.0)	0.026
Model 2	18.4 (17.3–19.4)	18.9 (18.4–19.4)	19.4 (18.8–19.9)	0.078
Model 3	18.3 (17.2–19.3)	18.9 (18.4–19.3)	19.4 (18.9–19.9)	0.038
AUCi (nmol l^−1 ^h^−1^)				
Model 1	1.13 (0.16–2.09)	2.38 (1.95–2.82)	2.38 (1.88–2.87)	0.106
Model 2	1.30 (0.34–2.27)	2.41 (1.97–2.84)	2.30 (1.80–2.79)	0.255
Model 3	1.21 (0.25–2.16)	2.41 (1.98–2.84)	2.32 (1.82–2.81)	0.190
Mean evening cortisol (nmol l^−1^)^a^				
Model 1	4.35 (4.01–4.71)	4.65 (4.48–4.81)	4.90 (4.71–5.11)^hc,ar^	0.004
Model 2	4.57 (4.24–4.93)	4.66 (4.50–4.82)	4.82 (4.63–5.00)	0.144
Model 3	4.57 (4.24–4.93)	4.66 (4.50–4.82)	4.81 (4.63–5.00)	0.151
Cortisol suppression ratio^a^				
Model 1	2.45 (2.27–2.65)	2.38 (2.29–2.46)	2.40 (2.31–2.50)	0.962
Model 2	2.40 (2.22–2.59)	2.37 (2.29–2.45)	2.43 (2.34–2.53)	0.492
Model 3	2.40 (2.22–2.59)	2.36 (2.28–2.45)	2.43 (2.34–2.53)	0.433
*Neurotrophic growth*	*n*=226	*n*=1204	*n*=1068	
BDNF (ng ml^−1^)				
Model 1	9.14 (8.71–9.56)	8.99 (8.81–9.17)	9.04 (8.84–9.24)	0.925
Model 2	9.21 (8.78–9.63)	9.01 (8.83–9.19)	9.00 (8.81–9.20)	0.544
Model 3	9.26 (8.83–9.70)	9.04 (8.85–9.22)	8.96 (8.76–9.16)	0.273
*Vitamin D*	*n*=228	*n*=1212	*n*=1074	
25(OH)D (nmol l^−1^)				
Model 1	71.4 (67.7–75.0)	64.5 (62.9–66.1)^hc^	59.8 (58.1–61.5)^hc,ar^	<0.001
Model 2	70.5 (66.9–74.1)	63.8 (62.3–65.4)^hc^	60.7 (59.1–62.4)^hc,ar^	<0.001
Model 3	70.7 (67.1–74.2)	63.7 (62.1–65.2)^hc^	60.9 (59.3–62.5)^hc,ar^	<0.001

Abbreviations: ar, at-risk for MDD group; AUCg/i, area under the curve with respect to the ground/increase; BDNF, brain-derived neurotrophic factor; CI, confidence interval; hc, healthy controls; HPA, hypothalamic–pituitary axis; MDD, major depressive disorder.

a Log-transformed factors presented back-transformed. Superscripts (^hc,ar^) refer to which group this group its outcome differs significantly (*P*<0.05) from; for example, the full-threshold MDD group has significantly lower vitamin D values in model 3 than both healthy controls and the at-risk for MDD group. Differences between groups were examined using Tukey's LSD *post hoc* tests. Model 1: adjusted for age; gender (except inflammation analyses, as these were stratified by gender); years of education. Model 2: additionally adjusted for alcohol status; smoking status; number of chronic diseases under treatment; body mass index. Model 3: additionally adjusted for covariates specific for the pathophysiological mechanism. Inflammation: systemic anti-inflammatory use. HPA-axis: awakening time, working status and season during saliva collection. BDNF: systemic anti-inflammatory use, non-opioid analgesic-antipyretic use and selective serotonin reuptake inhibitor use. Vitamin D: season during blood collection.

**Table 3:** Adjusted levels of pathophysiological markers across healthy controls and separate MDD stages

	*Healthy controls*	*At-risk for MDD stages*			*Full-threshold MDD stages*					*Trend analyses*, P*-values*		
*n =2563*	*HCs* n=*230* *mean (CI)*	*0* n=*287* *mean (CI)*	*1A* n=*116* *mean (CI)*	*1B* n=*834* *mean (CI)*	*2* n=*230* *mean (CI)*	*3A* n=*129* *mean (CI)*	*3B* n=*127* *mean (CI)*	*3C* n=*394* *mean (CI)*	*4* n=*216* *mean (CI)*	*HC and all stages*	*HC and at-risk for MDD stages (0, 1A and 1B)*	*Full-threshold MDD stages (2, 3A, 3B, 3C and 4*)
*Inflammation*	*n*=228	*n*=285	*n*=113	*n*=817	*n*=229	*n*=129	*n*=123	*n*=388	*n*=214			
*Male*	*n*=97	*n*=106	*n*=37	*n*=246	*n*=81	*n*=38	*n*=37	*n*=117	*n*=77			
C-reactive protein (mg l^−1^)^a^												
	1.02 (0.82–1.25)	1.01 (0.83–1.24)	1.11 (0.80–1.55)	1.03 (0.90–1.17)	1.43 (1.14–1.79)	1.31 (0.94–1.82)	1.06 (0.76–1.48)	1.42 (1.18–1.71)	1.10 (0.87–1.38)	*0.026*	0.691	0.312
Interleukin-6 (pg ml^−1^)^a^												
	0.68 (0.57–0.81)	0.84 (0.71–1.00)	0.86 (0.65–1.14)	0.79 (0.71–0.88)	0.97 (0.80–1.17)	0.85 (0.64–1.12)	0.69 (0.52–0.92)	0.95 (0.81–1.12)	0.80 (0.66–0.98)	0.102	0.279	0.467
*Female*	*n*=131	*n*=179	*n*=76	*n*=571	*n*=148	*n*=91	*n*=86	*n*=271	*n*=137			
C-reactive protein (mg l^−1^)^a^												
	1.40 (1.15–1.69)	1.50 (1.27–1.78)	1.59 (1.24–2.05)	1.37 (1.25–1.50)	1.40 (1.17–1.67)	1.49 (1.18–1.88)	1.18 (0.93–1.49)	1.21 (1.06–1.38)	1.30 (1.08–1.57)	*0.035*	0.538	0.237
Interleukin-6 (pg ml^−1^)^a^												
	0.79 (0.67–0.92)	0.81 (0.71–0.93)	0.76 (0.62–0.94)	0.71 (0.66–0.77)	0.77 (0.66–0.89)	0.84 (0.70–1.02)	0.61 (0.50–0.75)	0.73 (0.66–0.82)	0.66 (0.56–0.77)	0.090	*0.050*	0.177
*HPA-axis*	*n*=176	*n*=190	*n*=89	*n*=592	*n*=143	*n*=65	*n*=84	*n*=258	*n*=126			
AUCg (nmol l^−1^ h^−1^)												
	18.2 (17.2–19.3)	18.0 (17.0–19.0)	17.5 (16.0–18.9)	19.4 (18.8–19.9)^0;1A^	19.4 (18.2–20.5)^1A^	20.6 (18.8–22.3)^hc;0;1A^	19.1 (17.5–20.6)	19.5 (18.6–20.3)^0;1A^	19.0 (17.8–20.3)	*0.025*	*0.008*	0.446
AUCi (nmol l^−1^ h^−1^)												
	1.18 (0.22–2.13)	1.99 (1.05–2.92)	0.60 (−0.72–1.92)^0^	2.83 (2.30–3.35)^hc;1A^	2.15 (1.09–3.20)	2.94 (1.36–4.52)^1A^	2.46 (1.05–3.87)	2.15 (1.37–2.94)^1A^	2.50 (1.38–3.62)^1A^	0.150	*0.003*	0.793
Mean evening cortisol (nmol l^−1^)^a^												
	4.57 (4.24–4.93)	4.55 (4.24–4.90)	4.82 (4.34–5.36)	4.67 (4.48–4.86)	4.66 (4.29–5.06)	4.89 (4.31–5.53)	5.04 (4.52–5.61)	4.83 (4.54–5.14)	4.76 (4.37–5.21)	0.119	0.536	0.646
Cortisol suppression ratio^a^												
	2.40 (2.22–2.59)	2.36 (2.19–2.54)	2.65 (2.38–2.95)	2.32 (2.23–2.42)	2.61 (2.39–2.83)	2.32 (2.05–2.64)	2.20 (1.97–2.46)	2.39 (2.24–2.54)	2.57 (2.35–2.81)	0.651	0.396	0.693
*Neurotrophic growth*	*n*=226	*n*=282	*n*=113	*n*=809	*n*=226	*n*=125	*n*=123	*n*=385	*n*=209			
BDNF (ng ml^−1^)												
	9.25 (8.82–9.68)	8.75 (8.36–9.13)	8.69 (8.09–9.28)	9.18 (8.96–9.40)	9.08 (8.65–9.50)	8.84 (8.27–9.42)	9.01 (8.43–9.58)	8.94 (8.61–9.26)	8.98 (8.53–9.43)	0.628	0.499	0.828
*Vitamin D*	*n*=228	*n*=283	*n*=113	*n*=816	*n*=228	*n*=129	*n*=122	*n*=383	*n*=212			
25(OH)D (nmol l^−1^)												
	70.7 (67.2–74.3)	66.5 (63.3–69.7)	62.9 (57.9–67.8)^hc^	62.8 (60.9–64.7)^hc^	58.9 (55.4–62.4)^hc;0^	61.4 (56.7–66.1)^hc^	63.1 (58.3–67.9)^hc^	61.2 (58.5–63.9)^hc;0^	60.8 (57.1–64.4)^hc;0^	*<0.001*	*<0.001*	0.606

Abbreviations: AUCg/i, area under the curve with respect to the ground/increase; BDNF, brain-derived neurotrophic factor; CI, confidence interval; hc, healthy control; HPA, hypothalamic–pituitary axis; MDD, major depressive disorder.

a Log-transformed factors presented back-transformed. Superscripts (^1A,1B^) refer to which stage this stage its outcome differs significantly (*P*<0.05) from; for example, stage 4 has a significantly lower vitamin D value than both healthy controls and stage 0. Differences between groups were examined using Tukey's LSD *post hoc* tests. Adjusted for age; gender (except inflammation analyses, as these were stratified by gender); years of education; alcohol status; smoking status; number of chronic diseases under treatment; body mass index. Inflammation: systemic anti-Inflammatory use. HPA-axis: awakening time, working status and season during saliva collection. BDNF: systemic anti-inflammatory use, non-opioid analgesic-antipyretic use and selective serotonin reuptake inhibitor use. Vitamin D: season during blood collection.

**Table 4:** Associations between severity, duration, number of episodes and pathophysiological markers with the full-threshold MDD stages (stage 2 through 4).

	*Severity (IDS score)*			*Duration (% of time with depressive symptoms in 3 years before baseline)*			*Number of episodes*		
	*Beta*	*s.e.*	P*-value*	*Beta*	*s.e.*	P*-value*	*Beta*	*s.e.*	P*-value*
*Inflammation* *n*=1083	*n*=1064			*n*=1082			*n*=1048		
*Male*									
C-reactive protein (mg l^−1^)	−0.006	0.049	0.910	−0.029	0.047	0.534	−0.026	0.048	0.597
Interleukin-6 (pg ml^−1^)	0.063	0.055	0.255	0.005	0.053	0.927	0.053	0.055	0.338

*Female*									
C-reactive protein (mg l^−1^)	−0.031	0.035	0.376	0.006	0.034	0.847	−0.067	0.034	0.050
Interleukin-6 (pg ml^−^^1^)	0.020	0.038	0.599	−0.015	0.037	0.679	−0.028	0.037	0.456
*HPA-axis* *n*=676	*n*=617–617–669–636			*n*=619–619–672–639			*n*=600–600–653–621		
AUCg (nmol l^−^^1^ h^−^^1^)	−0.086	0.040	0.034	−0.059	0.039	0.128	−0.014	0.040	0.717
AUCi (nmol l^−^^1 ^h^−^^1^)	−0.002	0.042	0.956	0.020	0.040	0.613	−0.008	0.041	0.836
Mean evening cortisol (nmol l^−^^1^)	−0.046	0.037	0.215	−0.048	0.036	0.173	−0.026	0.036	0.482
Cortisol suppression ratio	−0.062	0.041	0.135	0.015	0.040	0.711	0.005	0.041	0.897
*Neurotrophic growth* *n*=1068	*n*=1050			*n*=1067			*n*=1033		
BDNF (ng ml^−^^1^)	−0.008	0.032	0.811	0.022	0.031	0.473	−0.018	0.032	0.562
*Vitamin D* *n*=1074	*n*=1055			*n*=1073			*n*=1039		
25(OH)D (nmol l^−^^1^)	−0.039	0.031	0.214	−0.022	0.030	0.458	0.034	0.030	0.252

Abbreviations: AUCg/i, area under the curve with respect to the ground/increase; BDNF, brain-derived neurotrophic factor; HPA, hypothalamic–pituitary axis; IDS, Inventory of Depressive Symptoms; MDD, major depressive disorder.

Clinical characteristics of MDD to predict measures of pathophysiological mechanisms, characteristics that together are used to create the staging model.

Adjusted for age; gender (except inflammation analyses, as these were stratified by gender); years of education; alcohol status; smoking status; number of chronic diseases under treatment; body mass index. Inflammation: systemic anti-Inflammatory use. HPA-axis: awakening time, working status and season during saliva collection. BDNF: systemic anti-Inflammatory use, non-opioid analgesic-antipyretic use and selective serotonin reuptake inhibitor use. Vitamin D: season during blood collection. Due to missing values in severity score, duration and number of episodes, the included number of participants is lower for these analyses. For example, our full-threshold MDD stages included 1074 participants with a vitamin D value; of those, 1055 had a severity score. Severity score was necessary to divide between stage 2 and 3A; however, those in stage 3B, 3C and 4 did not need a severity score to be staged.
