# Supplementary material for: Pathophysiology of major depressive disorder: mechanisms involved in etiology are not associated with clinical progression
Source: Transl Psychiatry. 2015 Sep 29;5(9):e649–. doi: 10.1038/tp.2015.137 (PMC5545638; doi:10.1038/tp.2015.137)
Supplement: Supplementary Information [file tp2015137x1.doc]

**Supplement Table 1: staging model for MDD**Criteria that were used to assign NESDA participants to MDD stages, and a short description of each stage (table based on Verduijn et al., 2015).^32^

|  |  | **DESCRIPTION** | **STAGE ASSIGNMENT CRITIRIA** |
| --- | --- | --- | --- |
| **AT-RISK for MDD STAGES** | **0** | Increased risk of depressive disorder; no symptoms currently. | - Never experienced an MDD or Anxiety disorder during life - IDS score ≤ 13 (none) - First degree family member with depressive disorder |
| **1A** | Increased risk of depressive disorder; mild or nonspecific symptoms of depression currently. | - Never experienced an MDD or Anxiety disorder during life - IDS score 14-25 (mild) |
| **1B** | Ultra-high risk of depressive disorder; moderate but sub-threshold symptoms of depression. | Group 1:   - Never experienced an MDD or Anxiety disorder during life - IDS score ≥ 26 (≥ moderate)   Group 2:   - MDD experienced during life, no current episode. |
| **FULL-THRESHOLD MDD STAGES** | **2** | Major depressive disorder, first episode. | - MDD current 1^st^ episode   when present in month prior to interview   - Symptom duration: short   when absent in month prior to interview   - IDS score ≤ 25 ( ≤ mild) - Symptom duration: short/medium |
| **3A** | Incomplete remission from first major depressive episode can proceed into stage 4 | - MDD current 1^st^ episode   when present in month prior to interview   - Symptom duration: medium   when absent in month prior to interview   - IDS score ≥ 26 ( ≥ moderate) - Symptom duration: short/medium |
| **3B** | Major depressive disorder, recurrent or relapsing episode after first episode. | - MDD current, second episode (or unknown number of recurrent episodes) - Symptom duration: short/medium |
| **3C** | Major depressive disorder: multiple relapses/reoccurrences. | - MDD current, multiple (≥3) episodes - Symptom duration: short/medium |
| **4** | Major depressive disorder, chronic. Note: could fast track to this stage at first presentation from stage 2 or from stage 3a | - MDD current, first or recurrent episode - Symptom duration: long |
| MDD = Major Depressive Disorder; MDD current = Major Depressive Disorder, 6 months recency; IDS = Inventory of Depressive Symptomatology, IDS severity categories from: [http://www.ids-qids.org/index2.html#table2](http://www.ids-qids.org/index2.html#table2), access date 2015-07-29.  Symptom duration:  Short = symptoms continuously present ≤ 6 months and present < 80% of the time in previous 3 years. Medium = symptoms continuously present > 6 - ≤ 24 months and present < 80% of the time in previous 3   years.  Long = symptoms continuously present > 24 months and/or present ≥ 80% of the time in previous 3 years. | | | |

Supplement Table 2: sample characteristics off healthy controls and separate MDD stages

|  | **HC** | **At risk for MDD stages** | | | **Full-threshold MDD stages** | | | | | **p-value** |
| --- | --- | --- | --- | --- | --- | --- | --- | --- | --- | --- |
| **n =2563** | **n=230** | **0**  **n=287** | **1A**  **n=116** | **1B**  **n=834** | **2**  **n=230** | **3A**  **n=129** | **3B**  **n=127** | **3C**  **n=394** | **4**  **n=216** |
| **Demographics** |  |  |  |  |  |  |  |  |  |  |
| Age (years), M (s.d.) | 43.5 (13.8) | 38.2 (15.3)^hc^ | 43.4 (13.9)^0^ | 43.6 (12.5)^0^ | 39.3 (12.2)^hc,1B^ | 38.2 (12.5)^hc,1B^ | 42.4 (12.1) | 41.1 (12.0)^1B^ | 42.7 (11.7)^0,3A^ | <.001 |
| Gender (female), n (%) | 132 (57.4) | 179 (62.4) | 78 (67.2) | 586 (70.3) | 149 (64.8) | 91 (70.5) | 88 (69.3) | 275 (69.8) | 137 (63.4) | .008 |
| Years of education, M (s.d.) | 13.2 (3.1) | 12.9 (3.2) | 11.9 (3.2)^hc^ | 12.4 (3.2)^hc^ | 11.6 (3.1)^hc,0,1B^ | 10.9 (3.1)^hc,0,1B^ | 11.2 (3.2)^hc,0,1B^ | 12.3 (3.2)^hc,3A,3B^ | 11.1 (3.3)^hc,0,1B,3C^ | <.001 |
| **Lifestyle and health factors** |  |  |  |  |  |  |  |  |  |  |
| BMI (kg m^-2^), M (s.d.) | 25.7 (4.6) | 24.5 (4.4) | 25.1 (4.6) | 25.7 (4.8)^0^ | 25.9 (5.6)^0^ | 25.9 (5.1) | 26.8 (5.5)^0^ | 25.2 (5.1) | 26.6 (5.9)^0^ | <.001 |
| Smoking status, n (%) |  |  |  |  |  |  |  |  |  | <.001 |
| Never | 92 (40.0) | 105 (36.6) | 40 (34.5) | 196 (23.5) | 67 (29.1) | 38 (29.5) | 33 (26.0) | 89 (22.6) | 63 (29.2) |  |
| Former | 88 (38.3) | 98 (34.1) | 42 (36.2) | 309 (37.1) | 63 (27.4) | 25 (19.4) | 39 (30.7) | 135 (34.3) | 50 (23.1) |  |
| Current | 50 (21.7) | 84 (29.3) | 34 (29.3) | 329 (39.4) | 100 (43.5) | 66 (51.2) | 55 (43.3) | 170 (43.1) | 103 (47.7) |  |
| Drinking behaviour, n (%) |  |  |  |  |  |  |  |  |  | <.001 |
| Non- drinker | 54 (23.5) | 67 (23.3) | 29 (25.0) | 240 (28.8) | 92 (40.0) | 61 (47.3) | 55 (43.3) | 138 (35.0) | 92 (42.6) |  |
| Mild-moderate drinker | 147 (63.9) | 193 (67.2) | 73 (62.9) | 497 (59.6) | 113 (49.1) | 58 (45.0) | 61 (48.0) | 208 (52.8) | 91 (42.1) |  |
| Heavy drinker | 29 (12.6) | 27 (9.4) | 14 (12.1) | 97 (11.6) | 25 (10.9) | 10 (7.8) | 11 (8.7) | 48 (12.2) | 33 (15.3) |  |
| Number of chronic diseases, M (s.d.) | 0.45 (0.71) | 0.42 (0.73) | 0.66 (0.97) | 0.62 (0.87)^0^ | 0.68 (0.90)^0^ | 0.73 (0.93)^0^ | 0.87 (1.13)^hc,0^ | 0.59 (0.88) | 0.77 (0.98)^hc,0^ | <.001 |
| **Pathophysiological mechanism markers and their specific covariates** | | |  |  |  |  |  |  |  | **p-value** |
| **Inflammation n=2526** | **n=228** | **n=285** | **n=113** | **n=817** | **n=229** | **n=129** | **n=123** | **n=388** | **n=214** |  |
| C-Reactive Protein (mg l^-1^)^a^, M (s.d.) | 1.14 (3.1) | 1.07 (3.2) | 1.31 (3.7) | 1.26 (3.4) | 1.51 (3.7)^0^ | 1.63 (3.7)^0^ | 1.34 (3.7) | 1.25 (3.5) | 1.43 (3.5) | .014 |
| Interleukin-6 (pg ml^-1^)^a^, M (s.d.) | 0.71 (2.5) | 0.73 (2.5) | 0.77 (3.3) | 0.74 (2.5) | 0.85 (2.5) | 0.88 (2.9) | 0.70 (2.9) | 0.78 (2.4) | 0.78 (2.9) | .261 |
| Systemic Anti-inflammatory med., n (%) | 2 (0.9) | 4 (1.4) | 6 (5.3) | 43 (5.3) | 9 (3.9) | 8 (6.2) | 7 (5.7) | 16 (4.1) | 10 (4.7) | .033 |
| **HPA-axis n=1723** | **n=176** | **n=190** | **n=89** | **n=592** | **n=143** | **n=65** | **n=84** | **n=258** | **n=126** |  |
| AUCg (nmol l^-1^ h^-1^), M (s.d.) | 18.2 (7.0) | 17.9 (6.2) | 17.5 (5.8) | 19.5 (6.7) | 19.0 (7.5) | 19.9 (8.0) | 19.0 (6.4) | 19.7 (7.7) | 19.1 (6.9) | .032 |
| AUCi (nmol l^-1^ h^-1^), M (s.d.) | 0.97 (6.5) | 1.98 (6.0) | 0.61 (6.3) | 2.80 (6.3)^hc^ | 2.11 (6.4) | 2.84 (7.4) | 2.57 (5.0) | 2.32 (6.2) | 2.58 (6.5) | .020 |
| Mean evening cortisol (nmol l^-1^)^a^, M (s.d.) | 4.33 (1.74) | 4.29 (1.74) | 4.66 (1.68) | 4.77 (1.69) | 4.65 (1.77) | 4.94 (1.79) | 5.10 (1.67) | 4.94 (1.77) | 4.92 (1.69) | .051 |
| Cortisol suppression ratio^a^, M (s.d.) | 2.45 (1.67) | 2.42 (1.62) | 2.70 (1.75) | 2.32 (1.61) | 2.58 (1.62) | 2.28 (1.88) | 2.16 (1.78) | 2.37 (1.61) | 2.51 (1.68) | .046 |
| Mean awakening time (h:min), M (s.d.) | 7.17 (1.08) | 7.25 (1.00) | 7.19 (0.57) | 7.29 (1.04) | 7.42 (1.14) | 7.39 (1.08) | 7.22 (1.09) | 7.26 (1.06) | 7.32 (1.13) | .038 |
| Working on day saliva collection, n (%) | 122 (69.3) | 127 (66.8) | 63 (70.8) | 376 (63.5) | 80 (55.9) | 29 (44.6) | 42 (50.0) | 163 (63.2) | 70 (55.6) | .001 |
| Season saliva collection (light), n (%) | 106 (60.2) | 105 (55.3) | 46 (51.7) | 396 (66.9) | 90 (62.9) | 45 (69.2) | 52 (61.9) | 147 (57.0) | 70 (55.6) | .010 |
| **Neurotrophic Growth n=2498** | **n=226** | **n=282** | **n=113** | **n=809** | **n=226** | **n=125** | **n=123** | **n=385** | **n=209** |  |
| BDNF (ng ml^-1^), M (s.d.) | 9.22 (3.01) | 8.51 (3.09) | 8.64 (3.33) | 9.24 (3.22) | 9.05 (3.52) | 8.81 (3.52) | 9.10 (3.15) | 8.93 (3.32) | 9.14 (3.54) | .085 |
| Systemic Anti-inflammatory med., n (%) | 2 (0.9) | 4 (1.4) | 6 (5.3) | 43 (5.3) | 9 (4.0) | 8 (6.4) | 7 (5.7) | 15 (3.9) | 10 (4.8) | .030 |
| Non-opioid analgesic-antipyretic med., n (%) | 16 (7.1) | 11 (3.9) | 10 (8.8) | 91 (11.2) | 19 (8.4) | 17 (13.6) | 14 (11.4) | 31 (8.1) | 26 (12.4) | .007 |
| Anti-depressant: SSRI, n (%) | 0 (0.0) | 2 (0.7) | 2 (1.8) | 127 (15.7) | 71 (31.4) | 48 (38.4) | 37 (30.1) | 83 (21.6) | 80 (38.3) | <.001 |
| **Vitamin D n=2514** | **n=228** | **n=283** | **n=113** | **n=816** | **n=228** | **n=129** | **n=122** | **n=383** | **n=212** |  |
| 25(OH)D (nmol l^-1^), M (s.d.) | 70.7 (27.4) | 68.3 (27.9) | 64.2 (27.4) | 63.2 (27.6)^hc^ | 57.8 (27.5)^hc,0^ | 60.3 (27.8)^hc^ | 61.6 (30.4) | 61.1 (28.1)^hc,0^ | 59.0 (29.5)^hc,0^ | <.001 |
| Season blood collection (light), n (%) | 123 (53.9) | 172 (60.8) | 62 (54.9) | 483 (59.2) | 123 (53.9) | 74 (57.4) | 72 (59.0) | 203 (53.0) | 12 (57.1) | .444 |

Abbreviations: AUCg/i , Area Under the Curve with respect to the ground/increase; BDNF, Brain Derived Neurotrophic Factor; BMI, Body Mass Index; hc, Healthy Controls; med=medication; M=Mean; MDD=Major Depressive Disorder; s.d.= standard deviation; SSRI=selective serotonin reuptake inhibitors.
^a^ Log-transformed factors presented back-transformed.
Superscripts (^hc,0^) refer to which stage this stage outcome differs significantly (p<0.05) from; for example, stage 4 is significantly older compared to stage 0, and stage 3A. Differences between groups were examined using Games-Howell post-hoc tests.
